# Genotypic and Phenotypic Resistance to Clarithromycin in *Helicobacter pylori* Strains

**DOI:** 10.3390/jcm9061930

**Published:** 2020-06-19

**Authors:** Eun Jeong Gong, Ji Yong Ahn, Jung Mogg Kim, Sun Mi Lee, Hee Kyong Na, Jeong Hoon Lee, Kee Wook Jung, Kee Don Choi, Do Hoon Kim, Ho June Song, Gin Hyug Lee, So Won Kim, Hwoon-Yong Jung

**Affiliations:** 1 Department of Internal Medicine, Gangneung Asan Hospital, University of Ulsan College of Medicine, Gangneung 25440, Korea; gong-eun@hanmail.net; 2 Department of Gastroenterology, Asan Medical Center, University of Ulsan College of Medicine, Seoul 05505, Korea; ji110@hanmail.net (J.Y.A.); hkna77@naver.com (H.K.N.); jhlee.gi@amc.seoul.kr (J.H.L.); jung.keewook30@gmail.com (K.W.J.); keedonchoi@gmail.com (K.D.C.); dohoon.md@gmail.com (D.H.K.); hjsong@amc.seoul.kr (H.J.S.); jhlee409@amc.seoul.kr (G.H.L.); 3 Department of Microbiology, Hanyang University College of Medicine, Seoul 04763, Korea; jungmogg@hanmail.net; 4 Asan Institute for Life Sciences, Asan Medical Center, Seoul 05505, Korea; eclipse-lsm@hanmail.net; 5 Department of Pharmacology, Asan Medical Center, University of Ulsan College of Medicine, Seoul 05505, Korea

**Keywords:** antibiotic resistance, *Helicobacter pylori*, whole-genome sequencing

## Abstract

Background: The increasing prevalence of antimicrobial resistance, together with the lack of novel treatment options, negatively affects successful eradication of *Helicobacter pylori*. The aim of this study was to investigate genetic mutations in the 23S rRNA genes, which is associated with clarithromycin resistance, and to determine the clinical impact of genotype on phenotypic antimicrobial resistance. Methods: A total of 46 *H. pylori* strains were obtained from 13 patients, before and after unsuccessful eradication with clarithromycin-based triple therapy. The phenotypic resistance of each *H. pylori* strain was determined by minimum inhibitory concentration against clarithromycin using the serial two-fold agar dilution method. The genomic sequences of 23S rRNA genes were identified through next-generation sequencing, and nucleotide variants were determined based on comparison with genome sequences of the reference strain *H. pylori* 26695. Results: Clarithromycin resistance was found in 9 of 13 subjects before treatment and all subjects after unsuccessful eradication. Whole-genome sequencing of the 23S rRNA genes detected 42 mutations on 40 nonidentical loci, including 2147A>G (formerly 2143A>G) and 2146A>G (formerly 2142A>G). All strains with clarithromycin-resistant phenotype had either 2147A>G or 2146A>G mutation. When comparing genotype and phenotype for clarithromycin resistance, there was a significant association between 2147A>G mutation and clarithromycin-resistant phenotype. Conclusions: All clarithromycin-resistant strains had either 2146A>G or 2147A>G mutation, suggesting that tests targeting these two mutations may be enough for the prediction of clarithromycin resistance in this population.

## 1. Introduction

*Helicobacter pylori* infection is the leading cause of chronic gastritis and is a major predisposing factor for peptic ulcer disease, mucosa-associated lymphoid tissue lymphoma, and gastric adenocarcinoma [1]. Since *H. pylori* was recognized as a pathogen, eradication therapy is assumed to have a potential role for prevention and treatment of *H. pylori*–associated gastrointestinal disorders [2]. Although the prevalence of *H. pylori* has decreased in recent decades, Korea is a region with a high prevalence of *H. pylori*; a nationwide investigation in 2015 and 2016 reported *H. pylori* seroprevalence of 51.0% [3]. Clarithromycin-based triple therapy for 7 to 14 days is recommended as the first-line treatment in Korea, and bismuth-based quadruple therapy may be considered where clarithromycin resistance is suspected to be over 15% [4]. However, the efficacy of first-line triple therapy has decreased in recent decades, with an increased prevalence of *H. pylori* strains with antimicrobial resistance, particularly against clarithromycin [5,6,7].

Since treatment for *H. pylori* infection is based on the empirical setting, it is essential to estimate the prevalence of regional antimicrobial resistance [8]. In addition, the importance of antimicrobial susceptibility testing has been emphasized to optimize eradication therapy. Indeed, tailored therapy based on the result of antimicrobial susceptibility testing showed superior outcomes over empirical treatment [9,10,11]. Nevertheless, data regarding antimicrobial resistance in Korea is limited.

Clarithromycin is a bacteriostatic antimicrobial agent that belongs to the macrolide. Point mutations in the peptidyl transferase region encoded in domain V of 23S ribosomal subunit (23S rRNA) are responsible for clarithromycin resistance. These mutations inhibit binding between clarithromycin and the ribosomal subunit dedicated to bacterial peptide translation [5]. Point mutations in one of two adjacent nucleotides in 23S rRNA, namely 2143A>G, 2142A>G, or 2142A>C, are the most commonly reported mutations in *H. pylori* with clarithromycin resistance [12,13,14,15]. Several other point mutations in the 23S rRNA are also reportedly involved in clarithromycin resistance [16,17]. However, the clinical relevance of these mutations remains controversial, and the association between genetic mutation and phenotypic antimicrobial resistance has not been fully investigated. In this study, we aimed to explore genetic mutations in the 23S rRNA genes and determine the clinical impact of genetic mutations on phenotypic antimicrobial resistance in *H. pylori* strains from Korean patients.

## 2. Materials and Methods

### 2.1. Culture and Antimicrobial Susceptibility Test

Retrospective analyses were performed on a total of 46 *H. pylori* strains prospectively collected from 13 subjects before and after unsuccessful clarithromycin-based triple therapy between January 2017 and September 2018. The duration of triple therapy was 7 days in all subjects. Two mucosal biopsy specimens were obtained from gastric antrum and corpus greater curvature, using standard-sized biopsy forceps during endoscopy. Samples were transported to the laboratory and maintained at room temperature, without transport media, until processing. Specimens were trimmed with a razor and streaked onto Brucella agar supplemented with 7% defibrinated sheep blood containing vancomycin (10 μg/mL), trimethoprim (5 μg/mL), amphotericin B (5 μg/mL), and polymyxin B (2.5 IU). The plates were incubated at 37 °C, under microaerophilic conditions for 5 to 7 days. After the initial isolation, colonies from each biopsy specimen were subcultured and identified as *H. pylori* based on their morphology and positive reactions with urease, oxidase, and catalase. All stock cultures were stored in Brucella broth supplemented with 15% glycerol and 10% fetal bovine serum at −80 °C. These preparations were thawed and subcultured for subsequent experiments. The minimum inhibitory concentration (MIC) of clarithromycin against *H. pylori* strain was determined by using the serial two-fold agar dilution method. The MIC value was defined at a minimum dilution concentration of the antimicrobial agent that did not produce bacterial colonies, and the resistant breakpoint of MIC was set at > 0.5 μg/mL for clarithromycin [18,19]. *H. pylori* strains from antrum and corpus specimens were evaluated separately. This study protocol was approved by the Institutional Review Board of Asan Medical Center (No. 2020-0518).

### 2.2. Detection of Mutations in the 23S rRNA Genes, Using Next-Generation Sequencing

Whole-genome sequencing of the 23S rRNA gene was performed on 46 *H. pylori* strains. For genome-sequencing assay, at least 1 μg of *H. pylori* genomic DNA was extracted, using a DNeasy Blood and Tissue kit, according to the manufacturer’s instructions (Qiagen, Hilden, Germany). Library preparation was performed by using the TruSeq^TM^ Nano DNA kit (Illumina Inc., San Diego, CA, USA) according to the manufacturer’ recommendation. Fragment size was estimated by an Agilent 2100 Bioanalyzer (Agilent Technologies, Santa Clara, CA, USA), with a DNA 1000 chip to visualize size distribution. Prepared libraries were quantified by using quantitative polymerase chain reaction according to the Illumina qPCR Quantification Protocol Guide. The estimate was further verified for size, using a 4200 TapeStation instrument with a D1000 ScreenTape (Agilent Technologies), and for quality by using Roche’s Rapid library standard quantification solution and calculator. The library was then pooled for Illumina MiSeq platform (Illumina, San Diego, CA, USA), using paired-end read for whole-genome resequencing.

Raw sequencing reads were filtered and mapped to the reference genome sequence of *H. pylori* strain 26695 (NCBI reference sequence: NC_000915), using BWA (version 0.7.17) with a mean coverage depth of 566-fold. After removing duplicates with Sambamba (version 0.6.7), single nucleotide variants and short indels were identified by analyzing the information obtained from aligned reads, using SAMTools (version 1.6) and SnpEff (version 4.3t). Candidate mutations in the 23S rRNA genes (*rrnA23S*) coding for the peptidyl transferase region of the 23S rRNA were identified by comparing the reconstructed genomes with those of the wild-type *H. pylori* strain 26695, which is susceptible to clarithromycin.

After comparing the entire genome sequences of the 23S rRNA gene of *H. pylori* for the reference strain (theoretical nucleotide length of 2975 bp, NC_000915.1:445249–448223) and that proposed by Taylor et al. (nucleotide length of 2967 bp) [20], mutation names were changed to conform with established sequence coordinates. That is, the nucleotide locations of mutations previously known as 2142 and 2143 correspond to locations 2146 and 2147 in the 23S rRNA genes of *H. pylori* strain 26695. The findings from this study depict the results as they were, and mutation names follow the previous format when citing previous studies.

### 2.3. Statistical Analysis

Representative values for continuous data are average values. The nominal data regarding association between genotype and phenotype of antimicrobial resistance were estimated by Fisher’s exact test. Significance was calculated as two-sided, and Bonferroni correction was used to resolve the multiple comparison issue. All analyses related to haplotype (frequencies and association with phenotype) were analyzed by using Haploview 4.2 software (Broad Institute, Cambridge, MA, USA).

## 3. Results

### 3.1. Prevalence of Antimicrobial Resistance to Clarithromycin

A total of 46 clinical *H. pylori* strains from 13 subjects were analyzed, including 22 primary strains (10 strains from gastric antrum and 12 strains from corpus) and 24 secondary strains obtained after eradication failure (11 strains from gastric antrum and 13 strains from corpus). Before treatment, 4 of 13 subjects had clarithromycin-susceptible strains with MIC distribution ranging from 0.0078 to 0.15 μg/mL, and nine subjects were determined to have resistant strains (MIC of 4 to 32 μg/mL). After unsuccessful eradication, all subjects showed clarithromycin-resistant phenotype, with MIC ranging from 8 to 128 μg/mL.

### 3.2. Mutations in the 23S rRNA Genes

**Figure 1:**
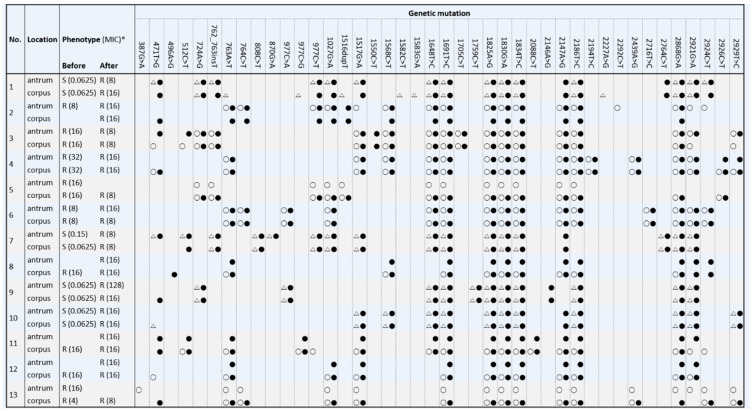
Phenotypic antimicrobial resistance to clarithromycin based on minimum inhibitory concentration (MIC) and genetic mutation in 23S rRNA genes of 46 *H. pylori* strains from 13 subjects, before (open symbol) and after (closed symbol) unsuccessful eradication therapy with clarithromycin-based triple therapy. Phenotypic resistance was classified as resistant (R, circle) or susceptible (S, triangle) based on MIC breakpoint of > 0.5 μg/mL. * Numbers in parentheses indicate MIC (μg/mL).

Comparison of reconstructed genome sequences with those of the reference strain revealed a total of 42 mutations on 40 nonidentical loci in the 23S rRNA genes (Figure 1 and Supplementary Figure S1). Four allele variations were found at location 977 (977C>T, A, or G), and each of these was analyzed separately. The number of mutations found within each strain ranged from 9 to 18, and no strains had single mutation. Among 42 mutations, 1691T>C, 1825A>G, 1830G>A, and 1834T>C were found in all patients regardless of antimicrobial susceptibility. In addition, 22 loci were shared among strains with susceptible and resistant phenotypes. Mutations of 387G>A, 1582C>T, 1583G>A, 2227A>G, and 2292C>T were observed only in primary strains and were considered variations that existed before the acquisition of clarithromycin resistance.

### 3.3. Genotypic and Phenotypic Resistance to Clarithromycin

Regarding phenotypic resistance, 29 mutations were found in clarithromycin-susceptible strains, and 39 mutations were found in clarithromycin-resistant strains (Supplementary Table S1). All 38 clarithromycin-resistant strains had either 2147A>G or 2146A>G mutation, equivalent to location 2143 and 2142 in domain V of the 23S rRNA genes proposed by Taylor et al. [20] The 2147A>G mutation was found in 94.7% (36/38) of strains with clarithromycin resistance and was observed in both primary and secondary strains with MIC ranging from 4 to 32 μg/mL (Table 1 and Supplementary Figure S2). The 2146A>G mutation was only found in two strains without 2147A>G.

Table 2 shows the association between allele frequencies and phenotypic antimicrobial resistance of each mutation in the 23S rRNA genes. The only significant difference in allele frequencies was observed for 2147A>G between clarithromycin-susceptible and -resistant strains. The association between 2147A>G and phenotypic antimicrobial resistance was found in 95.7% (44/46) of strains; the presence of G allele was associated with clarithromycin resistance in 94.7% (36/38) of strains, and A allele was associated with clarithromycin susceptibility in 100% (8/8) of strains.

## 4. Discussion

In the present study, we investigated the prevalence of mutations in the 23S rRNA genes conferring clarithromycin resistance, using next-generation sequencing of *H. pylori* strains. A total of 42 mutations, including 2146A>G and 2147A>G (formerly 2142A>G and 2143A>G), were found in 40 loci. All clarithromycin-resistant strains had either 2146A>G or 2147A>G mutation. That is, the combination of mutations 2146A>G and 2147A>G can predict resistant phenotype in all strains.

Clarithromycin is a cornerstone of the first-line eradication therapy for *H. pylori*, and the development of resistance to clarithromycin is a crucial factor for treatment failure [2,4]. Increasing antimicrobial resistance against *H. pylori* emphasizes the need for more precise and cost-effective methods to reliably predict antimicrobial resistance prior to eradication therapy. Currently, determination of antimicrobial susceptibility relies on culture-based methods performed on gastric biopsy tissue, which is time-consuming and requires a specific environment. Moreover, visual interpretations of MIC vary widely among individuals, which may lead to intra-and inter-observer variability. Various molecular methods, including next-generation sequencing, have enabled the detection of genetic mutations of *H. pylori* and suggested association between genotype and phenotype for antimicrobial resistance [21,22,23,24]. In the present study, whole-genome sequencing of the 23S rRNA gene was performed, and 42 mutations were detected. Each genotype was compared with phenotypic resistance to clarithromycin, and there was a clear association between the presence of 2147A>G mutation (formerly 2143A>G) and clarithromycin resistance.

The antimicrobial resistance mechanism of *H. pylori* is mainly based on point mutations in the bacterial chromosome [5]. Clarithromycin resistance is mediated by mutations in the 23S rRNA gene that reduce the affinity of clarithromycin for the 23S ribosomal component and inhibit clarithromycin activity against *H. pylori* [25]. The most commonly reported mutations include 2143A>G, 2142A>G, and 2142A>C [12,13,15,26,27]. Several other mutations have been recognized, such as 2182T>C, 2183T>C, 2223A>G, 2717T>C, 2245T>C, and 2116A>G (corresponding to nucleotide locations at 2186, 2187, 2227, 2722, 2249, and 2120 in this study), but their clinical relevance remains controversial [12,16,17,23,28,29,30,31,32]. Several reports suggested that 2143A>G is responsible for clarithromycin resistance and is associated with eradication failure [12,23,27,33,34]. In a previous study, 2143A>G mutation was found only in clarithromycin-resistant strains, and the presence of 2143A>G was correlated with the efficacy of eradication therapy [23]. Recent studies using whole-genome sequencing also found that clarithromycin resistance is associated with the presence of mutations 2146A>C (formerly 2142A>C), 2146A>G (formerly 2142A>G), or 2147A>G (formerly 2143A>G) in the 23S rRNA genes [15,22]. In the present study, clarithromycin resistance was primarily associated with 2147A>G, and clarithromycin-resistant strains without 2147G allele had the mutation 2146A>G. These results confirm that the 2147A>G mutation can be used as a reliable marker for clarithromycin resistance.

The prevalence of 2142A>G is reportedly lower than that of 2143A>G and is considered to be associated with clarithromycin resistance [13,15,26]. The impact of 2142A>G on outcomes of eradication therapy for *H. pylori* has been controversial. Previous studies showed a similar eradication rate for 2142A>G mutants and wild-type strains, while the rate significantly decreased with 2143A>G mutation [33,34]. According to a more recent report from the same group, the eradication rate of 2142A>G or 2142A>C mutants decreased to 57.1% compared to 96.4% of strains with genotypic and phenotypic susceptibility, suggesting that mutations at A2142 seemed to have a marginal role in conferring clarithromycin-resistant phenotype [35]. In contrast, other studies found that no strains with 2142A>G were eradicated by using clarithromycin-based triple therapy [36,37]. These discrepancies between studies may be due to the relatively low prevalence of 2142A>G, which makes it difficult to draw clear conclusions regarding the clinical impact of 2142A>G or 2142A>C mutations on eradication therapy. Although there were only two strains with 2146A>G mutation (formerly 2142A>G) in this study, which is not enough to obtain a statistically significant result, all strains with 2146A>G had clarithromycin-resistant phenotype. These results suggest that the presence of 2146G allele is associated with clarithromycin resistance. These findings should be further clarified in future studies.

The 2182T>C mutation was previously considered to be associated with clarithromycin resistance in *H. pylori* [17,29]. However, microbiologic evidence revealed that 2182T>C mutation was not responsible for clarithromycin resistance, which mainly depends on the presence of the 2143G allele [38]. The 2182T>C mutation was also frequently found in susceptible strains, as well as in resistant strains, and there was no association between the 2182T>C mutation and treatment outcome [23,31]. In this study, 2186T>C (equivalent to the location 2182 in the 23S rRNA genes, as proposed by Taylor et al. [20]) was found in 42 of 46 (91.3%) strains, regardless of phenotypic resistance. These results suggest that 2186T>C does not play a role in phenotypic resistance to clarithromycin. Linkage disequilibrium analysis showed no linkage disequilibrium between mutations at 2147 and 2186 (equivalent to the location 2143 and 2182), thus supporting these findings (Supplementary Table S2). In this study, however, the three indicators that define linkage disequilibrium (D’, LOD, and r-squared scores) were assumed to be double-stranded DNA, and all genotypes were calculated as homozygotes. Therefore, the linkage disequilibrium scores may be different with single-strand DNA of the bacteria.

This study has several limitations. First, confirmation that the mutations are involved in clarithromycin resistance by using transcriptional or functional analyses was not performed. To overcome this limitation, we compared each genotype with phenotypes determined by using a culture-based method to elucidate the clinical relevance of genotypic mutations in the 23S rRNA genes of *H. pylori.* Second, there were relatively few *H. pylori* strains, as it is difficult to obtain samples in pairs before and after eradication therapy in clinical settings. In particular, the results for 2146A>G mutation (formerly 2142A>G) may change if a sufficient number of cases are examined. Third, mutations in genes outside domain V of the 23S rRNA were not investigated. Recent studies reported mutations in multidrug efflux transporter genes involved in the development of resistance to clarithromycin [39]. In addition, mutations in other target genes, such as *rpl22* (encodes ribosomal protein that interacts with 23S rRNA domains) and *infB* (encodes translation initiation factor), have been shown to induce clarithromycin resistance, particularly in combination with mutations at A2146 and A2147 (equivalent to the location 2142 and 2143) [30]. Another possible limitation is that we determined genotypic resistance by using isolated strains rather than gastric biopsy tissue, and the prevalence of mixed infection was not considered. Despite these limitations, this study investigated genotypic resistance before and after clarithromycin-based triple therapy through whole-genome sequencing and suggested the clinical impact of mutations in 23S rRNA genes on phenotypic resistance, determined using agar dilution tests.

## 5. Conclusions

All clarithromycin-resistant strains had either 2146A>G or 2147A>G mutation (formerly 2142A>G and 2143A>G). These results suggest that testing for mutations at nucleotide positions 2146 and 2147 may be enough for the prediction of clarithromycin resistance in this population.

## Figures and Tables

**Table 1:** Phenotypic resistance to clarithromycin and alleles at nucleotide positions 2146 (formerly 2142) and 2147 (formerly 2143).

Strain No.	Phenotype	Allele at 2146	Allele at 2147	Strain No.	Phenotype	Allele at 2146	Allele at 2147
1	Susceptible	A	A	24	Susceptible	A	A
2	Susceptible	A	A	25	Resistant	A	G
3	Resistant	A	G	26	Resistant	A	G
4	Resistant	A	G	27	Resistant	A	G
5	Resistant	A	G	28	Resistant	A	G
6	Resistant	A	G	29	Resistant	A	G
7	Resistant	A	G	30	Susceptible	A	A
8	Resistant	A	G	31	Susceptible	A	A
9	Resistant	A	G	32	Resistant	G	A
10	Resistant	A	G	33	Resistant	G	A
11	Resistant	A	G	34	Susceptible	A	A
12	Resistant	A	G	35	Susceptible	A	A
13	Resistant	A	G	36	Resistant	A	G
14	Resistant	A	G	37	Resistant	A	G
15	Resistant	A	G	38	Resistant	A	G
16	Resistant	A	G	39	Resistant	A	G
17	Resistant	A	G	40	Resistant	A	G
18	Resistant	A	G	41	Resistant	A	G
19	Resistant	A	G	42	Resistant	A	G
20	Resistant	A	G	43	Resistant	A	G
21	Resistant	A	G	44	Resistant	A	G
22	Resistant	A	G	45	Resistant	A	G
23	Susceptible	A	A	46	Resistant	A	G

**Table 2:** Phenotypic antimicrobial resistance-related genotype distribution and allele frequencies.

Mutation *	Corresponding Allele	Frequency, *n* (%)	Mutant: Wild Type (Frequency, %)		*p*-Value
			Susceptible	Resistant	
387G>A (383)	A	1 (2.2)	0:8 (0)	1:37 (2.6)	>0.999
471T>G (467)	T	16 (34.8)	5:3 (62.5)	27:11 (71.1)	0.684
496A>G (492)	G	1 (2.2)	0:8 (0)	1:37 (2.6)	>0.999
512C>T (508)	T	8 (17.4)	1:7 (12.5)	7:31 (18.4)	>0.999
724A>G (720)	A	15 (32.6)	4:4 (50.0)	27:11 (71.1)	0.407
762_763insT (758)	No ins	14 (30.4)	5:3 (62.5)	27:11 (71.1)	0.684
763A>T (759)	T	24 (52.2)	1:7 (12.5)	23:15 (60.5)	0.020
764C>T (760)	T	10 (21.7)	0:8 (0)	10:28 (26.3)	0.171
808C>T (804)	C	4 (8.7)	6:2 (75.0)	36:2 (94.7)	0.134
870G>A (866)	G	2 (4.3)	7:1 (87.5)	37:1 (97.4)	0.321
977C>A (973)	C	8 (17.4)	6:2 (75.0)	32:6 (84.2)	0.613
977C>G (973)	C	4 (8.7)	7:1 (87.5)	35:3 (92.1)	0.548
977C>T (973)	C	13 (28.3)	5:3 (62.5)	28:10 (73.7)	0.669
1027G>A (1023)	A	23 (50.0)	3:5 (37.5)	20:18 (52.6)	0.700
1516dupT (1512)	TT	6 (13.0)	1:7 (12.5)	5:33 (13.2)	>0.999
1517G>A (1513)	A	29 (63.0)	5:3 (62.5)	24:14 (63.2)	>0.999
1550C>T (1546)	T	2 (4.3)	0:8 (0)	2:36 (5.3)	>0.999
1568C>T (1564)	T	18 (39.1)	2:6 (25.0)	16:22 (42.1)	0.453
1582C>T (1578)	C	1 (2.2)	7:1 (87.5)	38:0 (100.0)	0.174
1583G>A (1579)	G	1 (2.2)	7:1 (87.5)	38:0 (100.0)	0.174
1648T>C (1644)	T	37 (80.4)	0:8 (0)	9:29 (23.7)	0.324
1705C>T (1701)	T	4 (8.7)	0:8 (0)	4:34 (10.5)	>0.999
1759C>T (1755)	C	4 (8.7)	6:2 (75.0)	36:2 (94.7)	0.134
2088C>G (2084)	T	3 (6.5)	0:8 (0)	3:35 (7.9)	>0.999
2146A>G (2142)	G	2 (4.3)	0:8 (0)	2:36 (5.3)	>0.999
2147A>G (2143)	G	36 (78.3)	0:8 (0)	36:2 (94.7)	<0.001
2186T>C (2182)	C	42 (91.3)	6:2 (75.0)	36:2 (94.7)	0.134
2194T>C (2190)	C	4 (8.7)	0:8 (0)	4:34 (10.5)	>0.999
2227A>G (2223)	A	1 (2.2)	7:1 (87.5)	38:0 (100.0)	0.174
2292C>T (2288)	T	1 (2.2)	0:8 (0)	1:37 (2.6)	>0.999
2439A>G (2434)	G	7 (15.2)	0:8 (0)	7:31 (18.4)	0.325
2716T>C (2711)	C	4 (8.7)	0:8 (0)	4:34 (10.5)	>0.999
2764C>T (2759)	C	6 (13.0)	6:2 (75.0)	34:4 (89.5)	0.277
2868G>A (2860)	G	44 (95.7)	0:8 (0)	2:36 (5.3)	>0.999
2921G>A (2913)	G	36 (78.3)	0:8 (0)	10:28 (26.3)	0.171
2924C>T (2916)	T	14 (30.4)	1:7 (12.5)	13:25 (34.2)	0.403
2926C>T (2918)	T	5 (10.9)	0:8 (0)	5:33 (13.2)	0.569
2929T>C (2921)	C	13 (28.3)	2:6 (25.0)	11:27 (28.9)	>0.999

After Bonferroni correction, *p*-value of < 0.0012 is considered statistically significant. * Numbers in parentheses indicate equivalent nucleotide position proposed by Taylor et al. [20].

## Supplementary Materials

The following are available online at https://www.mdpi.com/2077-0383/9/6/1930/s1. Figure S1: Visually depicted location of mutations in 23S rRNA genes of 46 *H. pylori* strains. A total of 42 mutations were detected on 40 nonidentical loci; Figure S2. Mutations in 23S rRNA genes at each nucleotide position and distribution of minimum inhibitory concentrations (MIC) for clarithromycin before and after unsuccessful clarithromycin-containing triple therapy. Of note, 2147A>G mutation is found only in clarithromycin-resistant phenotype, with median MIC of 16 μg/mL (range, from 4 to 32 μg/mL). The 2186T>C mutation is detected in both clarithromycin-susceptible and resistant phenotypes. MIC, minimum inhibitory concentration; Table S1: Frequency of mutations in the 23S rRNA genes of 46 *H. pylori* strains according to the phenotypic resistance against clarithromycin before and after unsuccessful eradication therapy; Table S2. Assessment of linkage disequilibrium between mutations 2147A>G and 2186T>C in 23S rRNA genes.
