# Supplementary material for: Genotypic and Phenotypic Resistance to Clarithromycin in Helicobacter pylori Strains
Source: J Clin Med. 2020 Jun 19;9(6):1930. doi: 10.3390/jcm9061930 (PMC7356929; doi:10.3390/jcm9061930)
Supplement: Supplementary file 1 [file jcm-09-01930-s001.pdf]

Supplementary Table S1. Frequency of mutations in the 23S rRNA gene of 46 *H. pylori* strains according to the phenotypic resistance against clarithromycin before and after unsuccessful eradication therapy.

Position*	Susceptible (n = 8)	Resistant		
		Total (n = 38)	Primary resistance (n = 14)	Secondary resistance (n = 24)
387G>A (383)	0	1 (2.6)	1 (7.1)	0
471T>G (467)	3 (37.5)	13 (34.2)	3 (21.4)	10 (41.7)
496A>G (492)	0	1 (2.6)	0	1 (4.2)
512C>T (508)	1 (12.5)	7 (18.4)	2 (14.3)	5 (20.8)
724A>G (720)	4 (50.0)	11 (28.9)	4 (28.6)	7 (29.2)
762_763insT (758)	3 (37.5)	11 (28.9)	4 (28.6)	7 (29.9)
763A>T (759)	1 (12.5)	23 (60.5)	10 (71.4)	13 (54.2)
764C>T (760)	0	10 (26.3)	5 (35.7)	5 (20.8)
808C>T (804)	2 (25.0)	2 (5.3)	0	2 (8.3)
870G>A (866)	1 (12.5)	1 (2.6)	0	1 (4.2)
977C>T (973)	3 (37.5)	10 (26.3)	3 (21.4)	7 (29.2)
977C>A (973)	2 (25.0)	6 (15.8)	2 (14.3)	4 (16.7)
977C>G (973)	1 (12.5)	3 (7.9)	1 (7.1)	2 (8.3)
1027G>A (1023)	3 (37.5)	20 (52.6)	8 (57.1)	12 (50.0)
1516dupT (1512)	1 (12.5)	5 (13.2)	2 (14.3)	3 (12.5)
1517G>A (1513)	5 (62.5)	24 (63.2)	8 (57.1)	16 (66.7)
1550C>T (1546)	0	2 (5.3)	0	2 (8.3)
1568C>T (1564)	2 (25.0)	16 (42.1)	6 (42.9)	10 (41.7)
1582C>T (1578)	1 (12.5)	0	0	0
1583G>A (1579)	1 (12.5)	0	0	0
1648T>C (1644)	8 (100.0)	29 (76.3)	10 (71.4)	19 (79.2)
1691T>C (1687)	8 (100.0)	38 (100.0)	14 (100.0)	24 (100.0)

1705C>T (1701)	0	4 (10.5)	2 (14.3)	2 (8.3)
1759C>T (1755)	2 (25.0)	2 (5.3)	0	2 (8.3)
1825A>G (1821)	8 (100.0)	38 (100.0)	14 (100.0)	24 (100.0)
1830G>A (1826)	8 (100.0)	38 (100.0)	14 (100.0)	24 (100.0)
1834T>C (1830)	8 (100.0)	38 (100.0)	14 (100.0)	24 (100.0)
2088C>G (2084)	0	3 (7.9)	1 (7.1)	2 (8.3)
2146A>G (2142)	0	2 (5.3)	0	2 (8.3)
2147A>G (2143)	0	36 (94.7)	14 (100.0)	22 (91.7)
2186T>C (2182)	6 (75.0)	36 (94.7)	14 (100.0)	22 (91.7)
2194T>C (2190)	0	4 (10.5)	2 (9.1)	2 (8.3)
2227A>G (2223)	1 (12.5)	0	0	0
2292C>T (2288)	0	1 (2.6)	1 (7.1)	0
2439A>G (2434)	0	7 (18.4)	4 (28.6)	3 (12.5)
2716T>C (2711)	0	4 (10.5)	2 (14.3)	2 (8.3)
2764C>T (2759)	2 (25.0)	4 (10.5)	0	4 (16.7)
2868G>A (2860)	8 (100.0)	36 (94.7)	12 (85.7)	24 (100.0)
2921G>A (2913)	8 (100.0)	28 (73.7)	10 (71.4)	18 (75.0)
2924C>T (2916)	1 (12.5)	13 (34.2)	6 (42.9)	7 (29.2)
2926C>T (2918)	0	5 (13.2)	2 (14.3)	3 (12.5)
2929T>C (2921)	2 (25.0)	11 (28.9)	5 (35.7)	6 (25.0)

*Numbers in the parenthesis indicate equivalent nucleotide position proposed by Taylor et al.

Supplementary Table S2. Assessment of linkage disequilibrium between mutations 2147A>G and 2186T>C in 23S rRNA gene.

Haplotype	Frequency, %	Mutant:wild type (frequency, %)		*P* value
		Susceptible	Resistant	
GC	73.9	0:8 (0)	34:4 (89.5)	<0.001
AC	17.4	6:2 (75.0)	2:36 (5.3)	<0.001
GT	4.3	0:8 (0)	2:36 (5.3)	0.507

AT	4.3	2:6 (25.0)	0:38 (0)	0.002
D′ 0.361, LOD score 0.75, r^2 0.045				

D′, linkage disequilibrium coefficient; LOD, log of the odds.

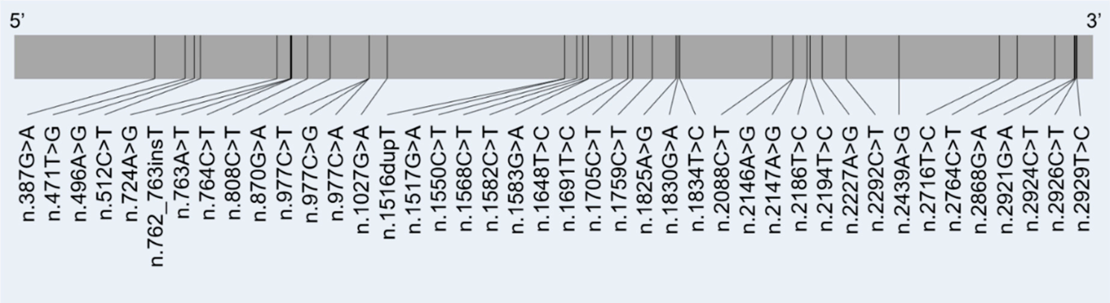

Supplementary Figure S1. Visually depicted location of mutations in 23S rRNA gene of 46 *H. pylori* strains. A total of 42 mutations were detected on 40 nonidentical loci.

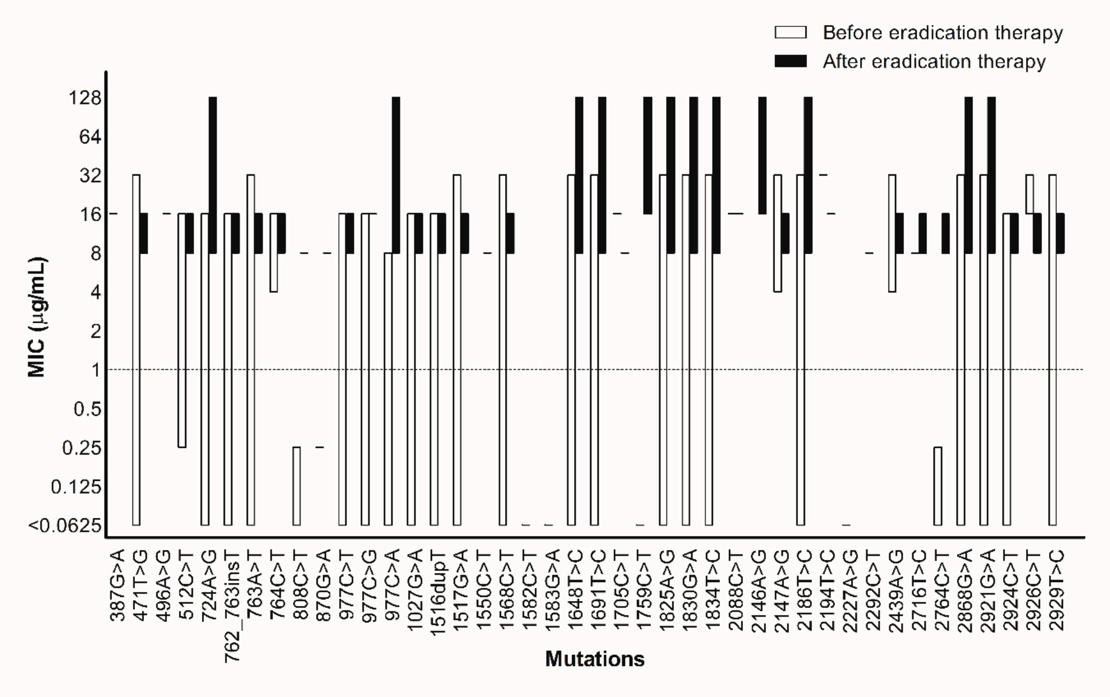

Supplementary Figure S2. Mutations in 23S rRNA gene at each nucleotide position and distribution of minimum inhibitory concentrations for clarithromycin before and after unsuccessful clarithromycin-containing triple therapy. Of note, 2147A>G mutation is found only in clarithromycin-resistant phenotype, with median MIC of 16 μg/mL (range, from 4 to 32 μg/mL). The 2186T>C mutation is detected in both clarithromycin-susceptible and resistant phenotypes. MIC, minimum inhibitory concentration.
